# webpic: A flexible web application for collecting distance and count measurements from images

**DOI:** 10.1371/journal.pone.0195184

**Published:** 2018-04-02

**Authors:** Lucy M. Chang

**Affiliations:** Department of Integrative Biology and Museum of Paleontology, University of California, Berkeley, California, United States of America; Baylor University, UNITED STATES

## Abstract

Despite increasing ability to store and analyze large amounts of data for organismal and ecological studies, the process of collecting distance and count measurements from images has largely remained time consuming and error-prone, particularly for tasks for which automation is difficult or impossible. Improving the efficiency of these tasks, which allows for more high quality data to be collected in a shorter amount of time, is therefore a high priority. The open-source web application, webpic, implements common web languages and widely available libraries and productivity apps to streamline the process of collecting distance and count measurements from images. In this paper, I introduce the framework of webpic and demonstrate one readily available feature of this application, linear measurements, using fossil leaf specimens. This application fills the gap between workflows accomplishable by individuals through existing software and those accomplishable by large, unmoderated crowds. It demonstrates that flexible web languages can be used to streamline time-intensive research tasks without the use of specialized equipment or proprietary software and highlights the potential for web resources to facilitate data collection in research tasks and outreach activities with improved efficiency.

## Introduction

Increasing demand for the collection and analysis of large amounts of raw data in the biological sciences has spurred numerous innovations in data synthesis and management [1] [2]. Morphological data, in particular, have been critical for understanding patterns in ecology and evolution, such as extinction selectivity [3], morphological divergence [4], and evolutionary relationships [5]. However, large-scale morphological datasets typically require a large investment of time and effort on the part of the researcher or many years of cumulative efforts across institutions. In general, the demand for large standardized morphological datasets outstrips the rate at which they can be collected, highlighting the need for fast and reliable data collection methods [6] [7].

As scanning and photographic technologies have improved and become more accessible, increased focus has been placed on developing new techniques in image analysis, enabling high-throughput capture of visual information. In particular, advances in automated image analysis have improved the speed at which visual features, such cell counts [8], can be processed, and completely machine-driven morphological character discovery and learning from images have begun to be implemented in tasks such as taxonomic identification, phylogenetics, and morphometrics [6] [9] [10]. Applying these approaches, however, often requires specialized software, hardware, or training, forcing researchers to overcome technical barriers before utilizing these powerful methods. There currently remains a need for ways to accommodate data collection workflows that require human judgment, such as the processing of inconsistent, low contrast, or difficult to characterize features.

Citizen or crowd-sourced science has become an increasingly popular solution to non-automated image processing, and many initiatives have successfully harnessed the interest and efforts of the public to process images on scales much larger than previously attempted [11] [12] [13]. Prominent examples of this include citizen science projects enabled through predominantly online platforms, such as Amazon Mechanical Turk (https://www.mturk.com/) and Zooniverse (https://www.zooniverse.org, e.g. Galaxy Zoo, Notes from Nature), taking advantage of the broad public accessibility that the internet provides. Though crowdsourcing has been shown to quickly provide accurate morphological data when compared to those generated by experts [14], no platform currently exists that is free, open-source, measurement-focused, and aimed at providing individual researchers crowdsource-like workflows for smaller-scale initiatives or outreach and education.

Current practices for non-automated image processing commonly include the use of software or equipment that may be costly to acquire licenses for or require specialized training to use. Simple browser-based tools can meet the broader need for non-automated approaches while possessing a number of other benefits not currently found in commonly used methods for collecting measurement data. These include but are not limited to:

1. Customizability. Almost any form of drawing and image manipulation may be implemented in a webpage, including freehand drawing, rotating, and recoloring. User-generated elements may additionally be forced to be dependent on one another. This degree of flexibility is especially useful when specimen measurements or counts require specific alignments and orientations. For example, user-generated points may be constrained to a line to guarantee that they are truly linear. The use of such constraints can serve to reduce measurement error.
2. Wide accessibility. Users may interact with the tool through an already familiar browser interface from any location with internet access and without the need for specialized programs or technical training. Additionally, data collected through multiple simultaneous and remote workflows can be automatically compiled into one easily shared location, encouraging collaborative use of data.
3. Increased transparency. An expansive array of data types and metadata can be recorded through web-based workflows. This includes angles, text input, timestamps, locations, and user identifiers. Such flexibility opens the doors for increased transparency and reproducibility by providing the means to pinpoint each decision made regarding the capture of information from the image.
4. Reduced sources of error. Because data within this framework are automatically stored in a format ready for analysis, it removes potential sources of error that come from recording and transferring measurements.
5. Cost. There is little to no cost involved with building, initiating, or using web-based tools nor do they require any special license. Resources and libraries for developing feature-rich websites are freely available, well-documented, and community-supported.

Here I present webpic, an open-source web application that enables images to be quickly measured and processed through an auto-advancing, streamlined workflow. I describe the framework of this application, including the resources used, its features, and its benefits. I then present a demonstration of the application using fossil leaf specimens, which I use to address the precision of browser-generated measurements in comparison to more commonly used methods.

## Materials and methods

### Web application components

**Fig 1:**
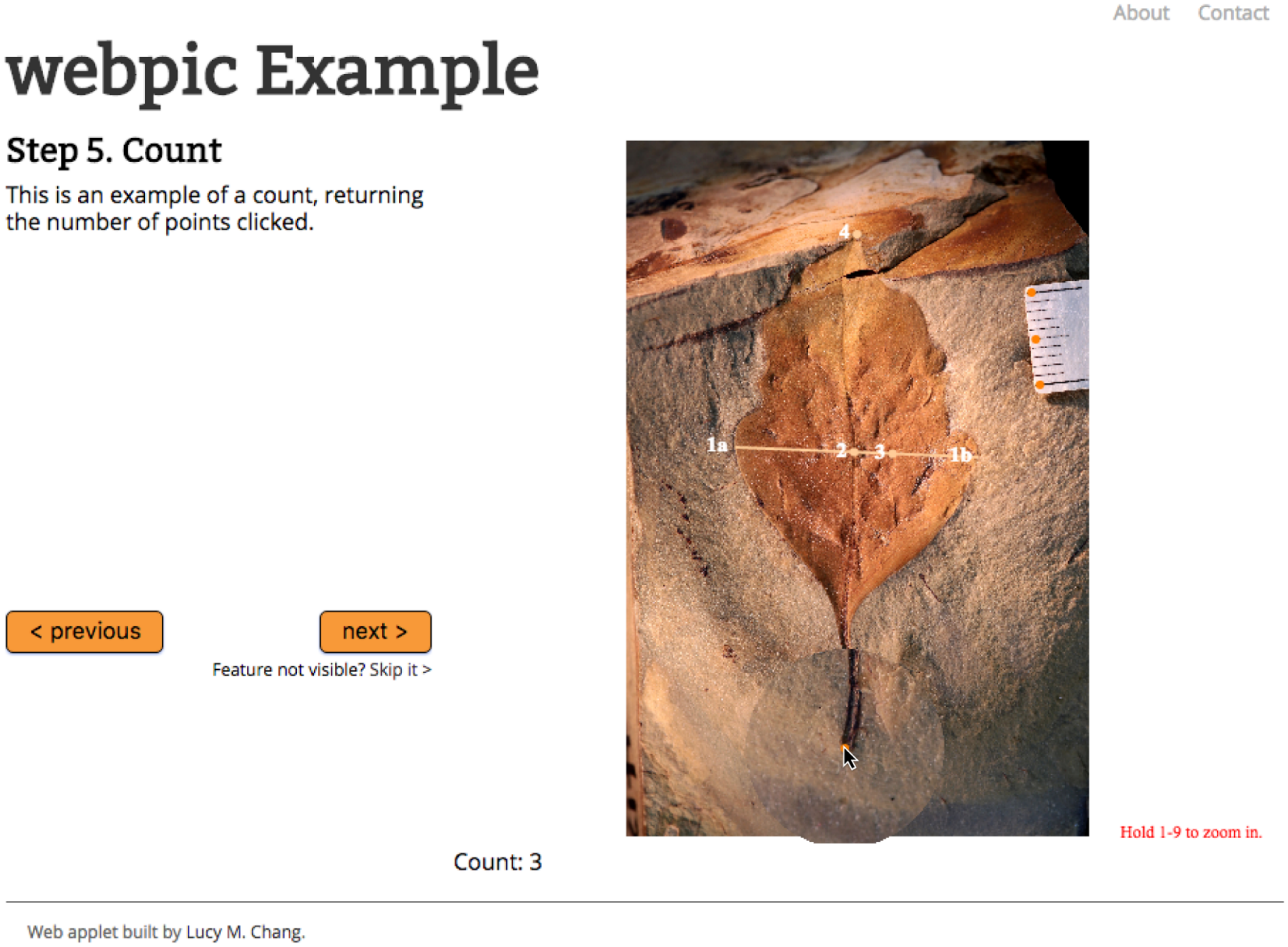
The web application, webpic, viewed in a browser window. Visual elements are labeled according to their associated step number. Currently supported visual elements include lines (here with endpoints marked “1a” and “1b”), points constrained to lines (“2”, “3”), unconstrained points (“4”), and counts (in darker orange). The zoom feature can be seen activated around the cursor.

webpic is an open-source web application (https://github.com/lucymchang/webpic) built using HTML5 and JavaScript libraries and, once hosted on a remote server, is accessible by the user through any web browser (Fig 1). This approach removes the need to download and train users in the use of specialized software. The application takes advantage of the canvas element in HTML5, which allows dynamic and interactive visual objects to be generated in the browser instantaneously. The application detects all images located in a designated folder and displays one to be processed in canvas, which the user interacts with through features implemented in Fabris.js (v1.5.0, http://fabricjs.com/), a freely available JavaScript library. The library includes functions that allow users to draw points and lines within canvas. In order to render these objects, absolute pixel positions in canvas are stored in JavaScript arrays. The application script then accesses these stored positions and uses them to calculate any desired measurements such as pixel distances.

With additional basic JavaScript, the measurements are fed directly into hidden input fields mirroring those of a pre-established Google Form (https://docs.google.com/forms/). Each field of the form is set up to receive one piece of information (e.g. a distance, position, or count). At the completion of the workflow, the stored measurements are submitted through the form, generating a record in an associated response table hosted by Google Sheets (https://docs.google.com/spreadsheets/). The web application then auto-advances to the next image. The researcher designates whether images are randomly pulled from the image folder or processed alphabetically by filename. Optional diagrams are similarly detected by the web application within the source folder and displayed at their corresponding steps. Optional integration with Google OAuth 2.0 can provide unique identifiers for data enterers, such as email addresses, for each submission. Additional user interface features are enabled using the jQuery JavaScript library (v2.1.3, https://jquery.com). Functionality of webpic at the time of publication includes: a persistent zoom feature to improve precision when measuring fine details; the option to constrain points along a drawn line; and the ability to return line lengths, distances between points and other elements, positions of points, and counts (see Table 1).

**Table 1:** Types of information that can currently be collected from images using webpic.

Supported data type	Value returned	Examples of use
image name	filename of the image being processed as a unique identifier (required)	
scale	scale factor used to scale the original image for rendering in canvas (required)	
line	pixel distance of a line drawn in canvas	measuring anatomical features or scale bars
point	position of the point or a distance to another point of line endpoint; point may be constrained to a line	placing landmarks or recording presences along a transect
count	count of points clicked	counting cells, pores, or zooids

Configuration of the workflow is managed by a single file, which outlines each step in JavaScript Object Notation (JSON). The application parses which data types to request from the user and populates customizable instructions for each step in the workflow. Because the programming of steps in the data collection process is modular, this framework is highly adaptable to diverse research questions.

### Web application generator

To minimize the barriers associated with implementing webpic for custom use, I created an additional interactive webpage that provides the necessary files for download and step-by-step instructions on how to generate a customized version of the application. To use the online generator, the researcher inputs the source code of the Google Form that will receive submitted data. The generator parses the source code and displays interactive tabs that the researcher uses to re-order, configure, and provide instructions for each step in the workflow. The final output from the generator is a string in JSON that is saved and uploaded along with the web application files and the images to be processed.

## Example of use

**Fig 2:**
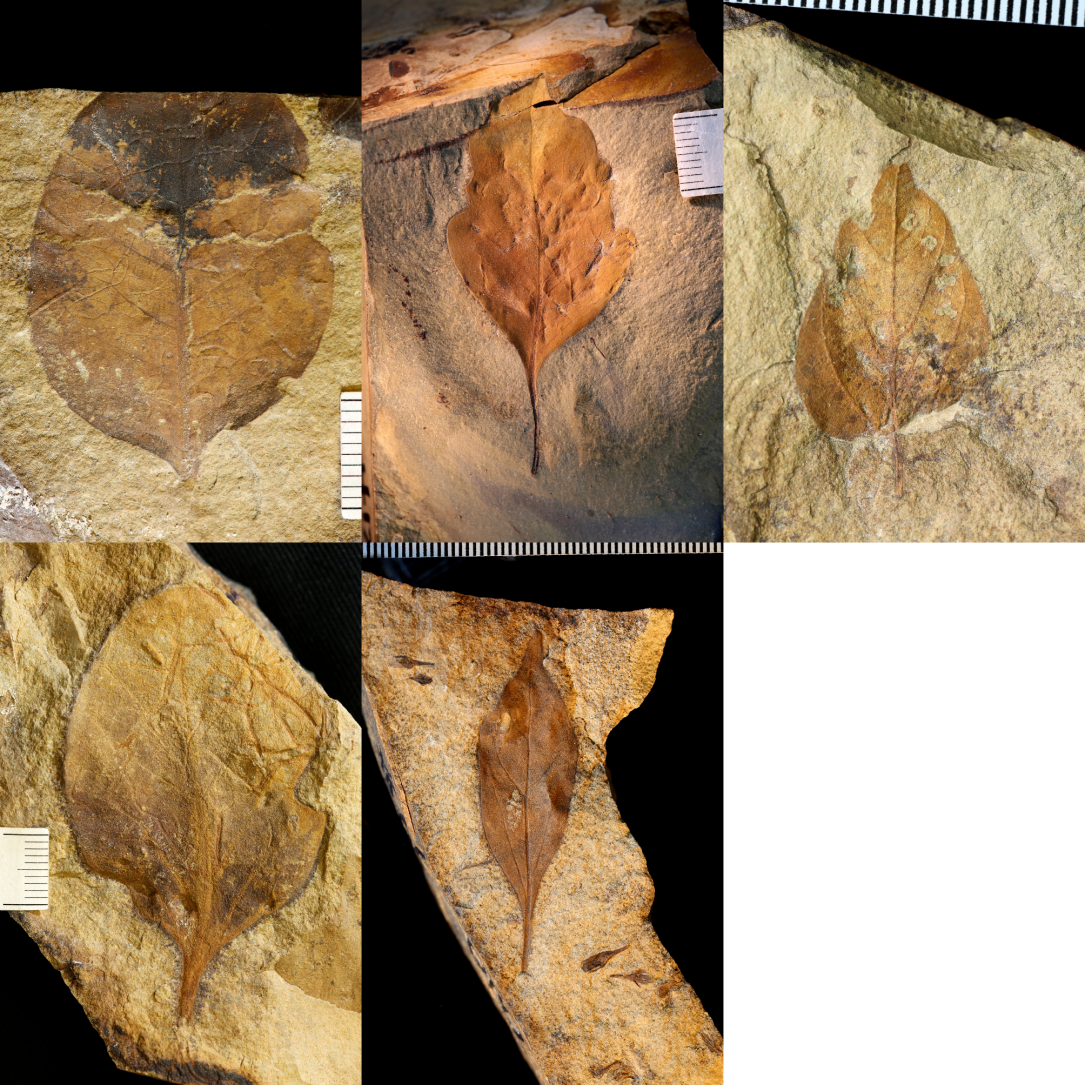
Images used to compare data collection methods. Specimens were collected from locality number TT92-3 (“Dori’s Tuff”). Specimens from top-left to bottom-right are: T827, T905, T907, T909, and X306.

To demonstrate the process of setting up and using webpic, five fossilized leaf specimens (Fig 2) were selected for processing and photographed at high resolution. Specimens used for this study are housed in the University of California Museum of Paleontology. Leaf shape analysis is useful for many ecological and evolutionary subjects including biodiversity, physiology, and paleoclimatic reconstructions, and improving methods for the digital capture of leaf morphology would facilitate these types of studies (see [9] for overview of common approaches). Various software programs have been developed to aid in the digital capture of leaf shape, either as standalone programs (e.g. SHAPE [15]) or for use in existing programs such as Image-J (e.g. LeafJ [16]). These programs are often highly tailored towards one or a few tasks and require training with new file formats and interfaces as well as access to a computer that houses the program and target files. webpic, on the other hand, provides a web-accessible interface and a framework within which functionality may be added while the overall step-by-step workflow and data submission process remains the same. Recent developments have improved methods of automation capable of processing increasingly more complex leaf features such as serration [17] or using machine learning algorithms trained on thousands of images to classify leaf features [18]. Though powerful, these methods often require high contrast images, advanced computing resources, or large datasets and thus may not be feasible for all intended cases, especially where specimen contrast may be poor or specimens are damaged or uncommon.

**Fig 3:**
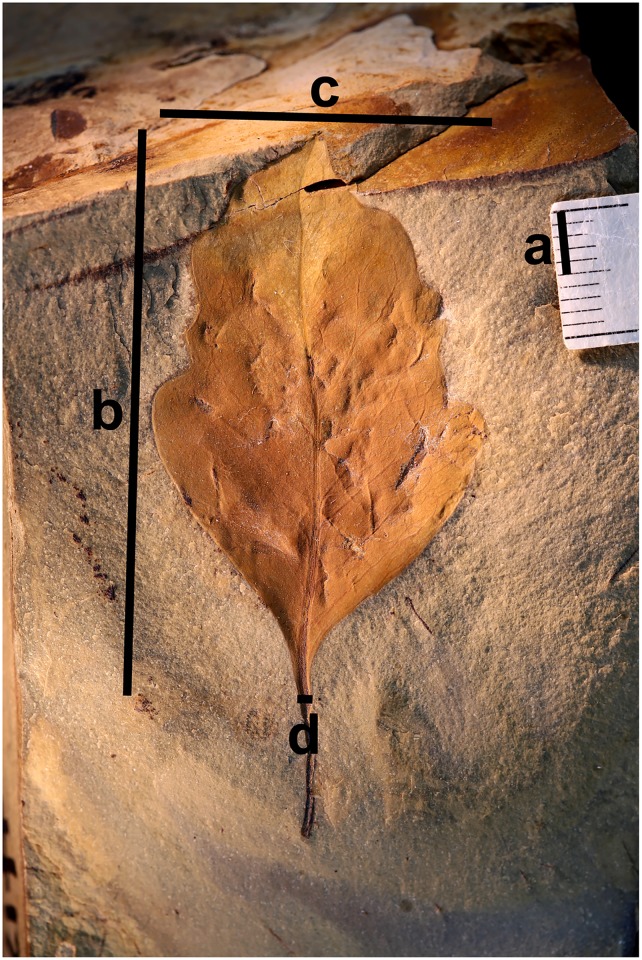
Photograph of an example fossil leaf specimen (T905) showing four linear measurements collected using different data collection methods. These measurements are: a) scale bar, b) leaf length, c) leaf width and d) petiole width.

To initiate the project, a Google Form was created containing six fields dedicated to capturing the following information submitted from webpic: the image filename, the scale factor used to display the image in the browser, the pixel length equivalent to five millimeters measured from the photographed scale bar, and the maximum length, width, and petiole width in pixels of the leaf (Fig 3). I then designated a data type for each step using the webpic generator and uploaded the JSON configuration file, web application files, and five specimen photographs (Fig 1) to a server.

To determine whether collecting measurements through a web browser interface is a suitable alternative to more traditional means of collecting similar data, I compared values obtained using the web application to those obtained from the same specimens using two commonly used methods in the biological and paleontological sciences: manually from original specimens using digital calipers and digitally using the measurement tool in a graphics editing software Adobe Photoshop (v13.0, see [19] for procedure).

Each specimen was measured twenty times with each of the three methods (S1 Table). The three methods used for comparison were chosen to reflect a range of approaches to studies that use linear measurements of morphology. Though other types of measurement are available in webpic (see Table 1), linear measurements are the most sensitive to imprecision and error because they measure a continuous variable and incorporate the error from both of its two endpoints. Assessment of linear measurements would thus best reveal any major weaknesses with using web-based applications to collect measurements.

**Fig 4:**
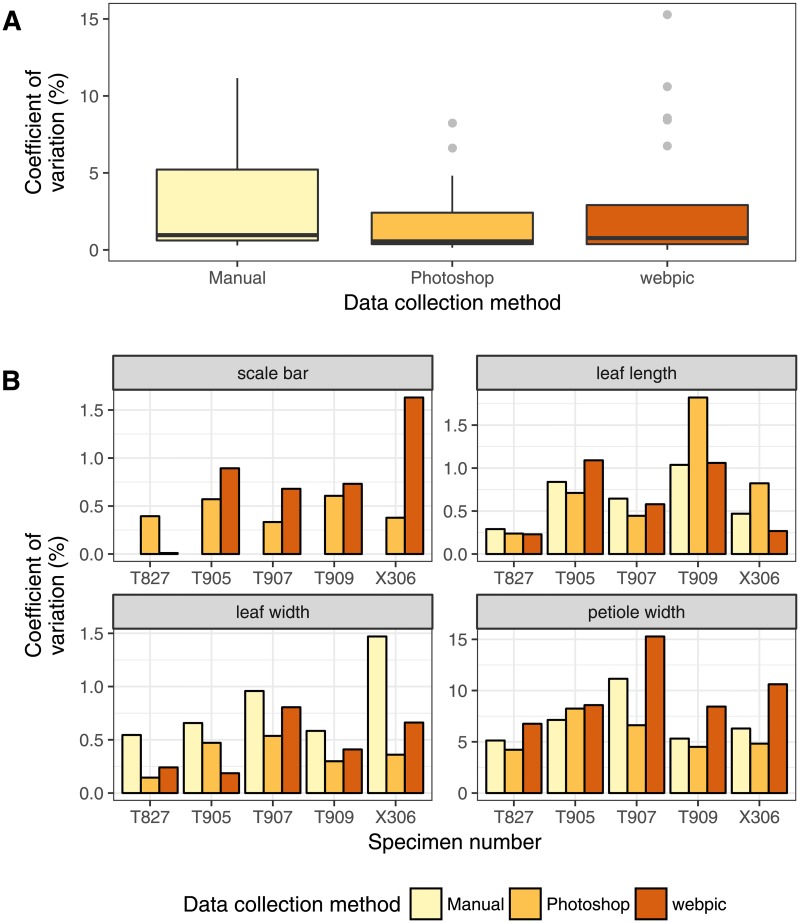
Coefficients of variation (CV) obtained using each data collection method across four leaf measurements. A: Combined CV is shown for each method. Boxes denote interquartile ranges (IQR), whiskers encompass data within 1.5 IQR beyond the first and third quartiles, and points indicate outliers. B: CV values of the four different measurements for each specimen.

To evaluate the precision of web-based measurements taken using webpic, I calculated the coefficient of variation (CV) for the twenty replicates of each measurement taken from each specimen in the fossil leaf demonstration (Fig 4A). CV values, which provide the ratio of the standard deviation to the mean as a percentage, are useful in this case, as the units of distance are not directly comparable between the three methods. I found no significant difference between the distributions of CV values for each data collection method (Kruskal-Wallis test, *p* = 0.26), suggesting no one method is more precise than the others.

When parsed out by measurement for individual specimens (Fig 4B), the overall shortest measurement, petiole width, contributed the highest CV values in all three methods by an order of magnitude. This suggests that the largest source of error among all of the presented methods lies in the user’s ability to discern details given the image’s resolution. Measurements obtained of the two shortest lengths, scale bar and petiole width, using Photoshop or by hand were overall less variable than those taken using webpic, where these lengths measure less than 50 pixels. For the two longest lengths, leaf length and leaf width, webpic was as precise than the other two methods or, as in the case of leaf width, consistently more precise. Overall, CV values for the three longest measurements were low, not exceeding 2% for any data collection method.

**Fig 5:**
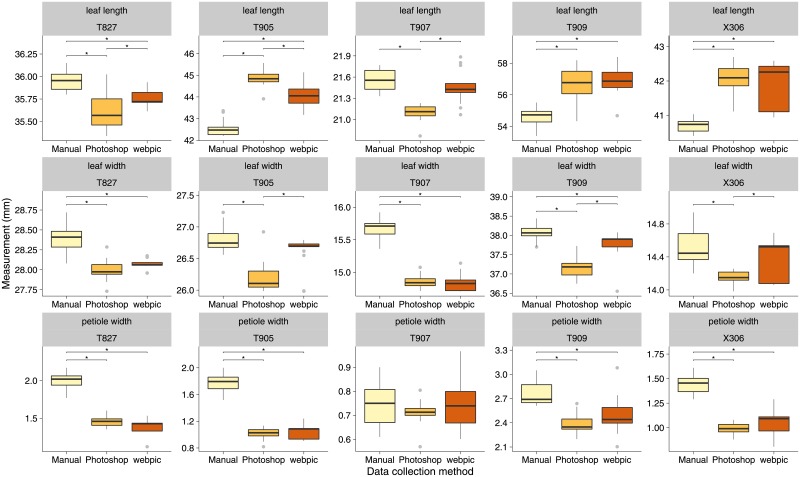
Measurements obtained using the three data collection methods for all five specimens following conversion from pixels to millimeters. Asterisks indicate significant differences (*p* < 0.05) in means using Tukey’s HSD test with *p*-value adjustment.

I subsequently examined whether the three data collection methods obtained similar estimates of distance. Lengths in millimeters were calculated for data collected using Photoshop and webpic by dividing the measured lengths in pixels by the pixel length corresponding to five millimeters on the photographed scale bars (Fig 5). This conversion compounds the error from two measurements but is meant to simulate the application of these methods in real-life workflows. Pairwise comparisons of observed means for each measurement and specimen (Tukey’s HSD test, see S2 Table for results) do not indicate consistent biases in significance and direction of differences between measurements obtained using webpic and those obtained using the other two methods. Photoshop and webpic returned similar values for the shortest measurement, petiole width. These values were often smaller than those obtained though manual measurement. The difference between digital and manual data collection methods can be seen in other measurements as well, though the direction and magnitude of the difference varies in the longer measurements. The three data collection methods frequently recovered significantly different estimates of the measured lengths (Fig 5).

## Discussion

Modern studies in biology and paleontology increasingly generate and use large amounts of morphological data to understand the ecological and evolutionary underpinnings of phenotypic change across space and time. Increasing the efficiency and reliability of collecting these data, however, remains an ongoing challenge, and we are only in the early stages of using modern technologies and global accessibility to meet this challenge. The application webpic and provided example of use suggest that with thoughtful design and assessment, web-based tools may provide a powerful alternative to currently used methods for collecting morphological data, allowing individual researchers to mobilize high-throughput data collection efforts.

Though the provided example focuses on fossil leaf shape measurements, webpic is broadly adaptable to research goals in and out of the biological sciences that require collection of relatively simple data from images. For example, the feature that allows points to be constrained to a line can be used to collect ecological data such as the distance between organisms along a transect, and the count feature has been used to document the number of kelp propagules on photographed plates. webpic is best suited for tasks involving repetitive processing of low contrast images or highly irregular shapes as well as other tasks that are difficult to automate and require human judgment. Additional improvements can be made to its functionality, such as adding support for more complex measurements and text input.

In the fossil leaf example presented here, I find no significant difference overall between the precision of webpic compared to other data collection methods across all measurements. At very short linear measurements, the manual approach and Photoshop (CV values ranging between 5–11% and 4–8%, respectively, for petiole width) showed slightly greater precision than webpic (CV values ranging between 7–15%). However, lowered precision across all methods for shorter lengths compared to that of longer lengths indicates image quality may be a more limiting factor for collecting accurate measurements than the collection method used.

On top of the potential for web-based applications to compete with existing methods in completing identical tasks, there are unique benefits to the use of this method, such as wide accessibility to files and the workflow, the ability to run concurrent instances and to collect metadata, and automatic submission to a centralized, collaboration-ready data repository. The result is a data collection workflow with increased transparency and a reduction in the number of potential sources of error that arise from bookkeeping or lack of organization.

It is always recommended that the data quality collected through customized web workflows be assessed before full implementation. However, by prioritizing free and highly adaptable resources, web-based tools such as the one presented here have the potential for broad applications, streamlining and disseminating tasks both in research, including citizen science, and in education, including outreach and training.

## Supporting information

S1 Fig Screenshots showing each step in building a custom webpic site. (1) Creation of a Google Form. (2) Configuration of workflow in the webpic generator. (3) Image processing via webpic. (4) Retrieval of submitted data in Google Sheets.
(TIF)

S1 Table Measurements used to compare data collection methods. (CSV)

S2 Table Results from Tukey’s HSD tests for differences in observed mean measurements between data collection methods following unit conversion. Table includes the difference in the observed means, the lower and upper bounds of the 95% confidence interval, and the *p*-value adjusted for multiple comparisons.
(CSV)

S1 Appendix R code used for analyses and generation of figures and supporting table related to the provided example of use. (R)
